# Transcriptome Analysis of *TGFBI* Knockdown vs Normal Corneal Epithelial Cells: Implications for *TGFBI* Corneal Dystrophy Treatment

**DOI:** 10.1007/s10528-025-11191-3

**Published:** 2025-07-15

**Authors:** Gabriella Guo Sciriha, Josef Borg, Janet Sultana, Joseph Borg

**Affiliations:** 1 Faculty of Medicine and Surgery, University of Malta, Msida, Malta; 2 Department of Ophthalmology, Faculty of Medicine and Surgery, Mater Dei Hospital, Msida, Malta; 3 Department of Applied Biomedical Science, Faculty of Health Sciences, University of Malta, Msida, Malta; 4 Department of Pharmacology and Clinical Therapeutics, Faculty of Medicine and Surgery, University of Malta, Msida, Malta; 5 Exeter School of Medicine and Health, University of Exeter, Exeter, UK

**Keywords:** Transforming Growth Factor Beta Induced, Corneal dystrophy, RNA interference

## Abstract

This study aimed to clarify the role Transforming Growth Factor Beta Induced (*TGFBI*) protein plays in corneal epithelial homeostasis by using RNA interference and to explore the possibility of gene therapy as a treatment modality for the visually debilitating *TGFBI* Corneal Dystrophies (CDs). *TGFBI* knockdown (KD) in Human Corneal Epithelial Cells (HCECs) was performed by using shRNA lentiviral vectors. RNA sequencing and comprehensive transcriptome analysis were performed to investigate the differential expression between control HCECs and *TGFBI* KD HCECs. Over Representation Analysis of the differentially expressed (DE) genes delineated the effect inhibition of *TGFBI* would have on molecular pathways, corneal structure and function. An effective KD of 70.5% was achieved. The functions of downregulated genes in *TGFBI* KD HCECs indicate decreased inflammation (*MTPN, IL1B, IL6, JAK2*), decreased angiogenesis (*CD24, IL6, JAK2*), and decreased corneal scarring (*AREG, ITGA11*). The functions of upregulated genes indicate increased ECM remodeling, fibrosis, and neovascularisation (*MMP2, AKT1, COL6A1, COL6A2*), increased integrin signaling (*ICAM, ITGA6*), and increased cell proliferation (*AKT1, ITGA6*). Enriched associations of the DE genes included cell adhesion molecules, ECM structural constituents, RNA transport & metabolism, SMAD2/SMAD3:SMAD4 modulation, JAK-STAT and PI3K-Akt signaling pathways. This proof-of-concept study shows that it is possible to effectively silence *TGFBI* with shRNA in HCECs and provides valuable insight into how *TGFBI* dysfunction might impact corneal epithelial function. In view of the lack of targeted treatment available, the therapeutic potential of shRNA targeting *TGFBI* should be explored further since it can potentially revolutionize the future management of *TGFBI* CDs.

## Introduction

The majority of cells in an organism have exactly the same genome. Nevertheless different types of cells exist. This is because a cell usually expresses only a fraction of its genes, with each cell type expressing a different set of genes, making it unique. The gene expression pattern of the cornea is mirrored in the unique characteristics of this avascular and serum-free tissue. When, how and which genes are expressed, in order for the appropriate proteins to be synthesized, contributes to the proper functioning of the cell (SHen 2023). Research focusing on the cornea’s unique properties will help understand the role certain genes play in the homeostasis of this tissue.

When the cell’s physiological balance is tipped due to genetic mutations or alterations in gene expression, a variety of disorders can arise. These gene alterations can potentially have various downstream effects on other genes involved in various cellular processes. Thus, analyzing the physiological role certain genes have in a particular cell type will lead to the understanding of the pathophysiology involved in the clinical manifestations of diseases such as the genetically inherited corneal dystrophies (CDs). CDs are a group of genetic conditions characterized by non-inflammatory, bilateral, often progressive alterations of the cornea (Weiss et al. 2024, 2015). CDs are considered to be rare, and research on their worldwide prevalence is ongoing (Guo Sciriha et al. 2022). In CDs, corneal opacification occurs due to deposition of abnormal material in the cornea that in turn leads to decompensation or scarring. Since the cornea is a clear tissue, deposition of abnormal material compromises its normal functionality with consequent decrease in vision (KLintworth 2009; Bron 1990; Glavini 2013).

Epithelial–Stromal *TGFBI* CDs is a subgroup of CDs where mutations in the *TGFBI* gene, which is located on chromosome 5q31, are responsible for a number of phenotypically heterogeneous CDs that affect multiple layers of the cornea (Weiss et al. 2024; Han et al. 2016). In the human eye, *TGFBI* is transcribed almost exclusively in the corneal epithelium and to a lesser extent in the stromal keratocytes. Once secreted, TGFBIp diffuses from the epithelium into the underlying stroma where it contributes to the functional and structural integrity of the cornea, which relies on the specific and orderly interaction of the constituting macromolecular components in the extracellular matrix (ECM) (Hartz and McKusick 2015; Stenvag et al. 2013). Notably, approximately 60% of human corneal TGFBIp (65 kDa) is covalently associated with insoluble ECM components (Runager et al. 2013; Andersen et al. 2004). Such stable interactions could be important for the biological functions of ECM and TGFBIp itself.

The human *TGFBI* gene codes for a 683 amino acid protein, named TGFBIp, which is an extracellular matrix protein that is expressed ubiquitously in multiple human tissues and is highly conserved between species. TGFBIp consists of a secretory signal peptide sequence, an N-terminal EMI (CROPT) domain (found originally in the EMILIN-1 protein), four fasciclin-like homologous internal repeat domains (FAS1 domains), and a COOH-terminal Arg-Gly-Asp (RGD) sequence which binds to integrin. The EMI is a cysteine rich domain that is present in many human proteins. Its function is not yet confirmed, however, it is thought that it mediates protein-protein interactions (Callebaut et al. 2003). The RGD motif, which has been identified in TGFBIp, is known to be present in many extracellular matrix proteins. It plays a role in the binding of TGFBIp to collagen, to the glycoproteins laminin and fibronectin as well as to the proteoglycans biglycan and decorin (Thapa et al. 2007; Kim et al. 2002).

Collagens form a large part of the corneal ECM and play an important role in the maintenance of corneal clarity. TGFBIp binds via noncovalent bonds to collagen types I, II, and IV (Nielsen et al. 2020; Runager et al. 2013; BilLings et al. 2002). On the other hand, collagen type VI forms both noncovalent and covalent connections with TGFBIp (Kim et al. 2002; Reinboth et al. 2006; Hanssen and Reinboth 2003). More than half of the TGFBIp present in the human cornea is covalently bound to collagen type XII by a reducible bond, making it insoluble (Nielsen et al. 2020; Andersen et al. 2004). Literature has revealed that TGFBIp is involved in physiologic and pathologic pathways by mediating cell proliferation, differentiation, adhesion (Maeng et al. 2017; Park et al. 2009; Skonier et al. 1992) and migration (Park et al. 2021; Nam et al. 2006). While these interactions most likely play a crucial role in ECM function, the physiological function of TGFBIp in the cornea has yet to be understood fully (CHen et al. 2020).

Multiple researchers confirmed that the deposits present in corneas of TGFBI-linked CDs are composed of aggregates largely containing TGFBIp (CHang et al. 2024; Karring et al. 2012; Courtney et al. 2015). However, even though mutations in *TGFBI* gene have been strongly linked with certain corneal dystrophy subtypes, the way the mutated TGFBIp causes corneal deposits still remains unknown. Various hypotheses exist regarding the cause of mutated TGFBIp deposits in TGFBI-linked CDs with proposals varying according to the type of mutation present. Some state that it is concentration-dependent while others postulate that certain *TGFBI* mutations affect protein-protein interactions directly while other mutation variants cause misfolding of the protein and altered TGFBIp stability and solubility (Kim et al. 2022; Venkatraman et al. 2020; Clout and HoHenester 2003). Additionally, other proposals focused on the increased susceptibility of fibroblasts to oxidative stress, and the possibility of a defective autophagy leading to altered clearance of the mutant deposits (Jun et al. 2025; Choi et al. 2012). However, fascinatingly, even though mutated TGFBIp is produced by cells in many organs in the body, deposits have only been located in the cornea (Runager et al. 2008; Kochairi et al. 2006). This leads to the postulation that for corneal deposits to occur in *TGFBI* CDs, a unique extracellular environment in the cornea is necessary (Nielsen et al. 2020; Kochairi et al. 2006; Kim et al. 2002).

Presently, there is no disease-modifying treatment for *TGFBI* CDs. The only treatment available for the management of *TGFBI* CD patients consists of supportive medical treatment to relieve discomfort, and superficial keratectomy, laser phototherapeutic keratectomy or corneal graft surgery to treat visual loss. Unfortunately, each surgical technique can be associated with a number of serious complications, such as graft rejection in corneal graft surgery. Furthermore, recurrence of the dystrophy within the graft has been reported to be almost universal within a few years (Chaurasia et al. 2023; Lyons et al. 1994). As a result, patients might have to undergo multiple procedures over a lifetime due to recurrence of the dystrophy, resulting in a poor quality of life for these individuals. Thus, ideally, the management of *TGFBI* CDs should revolve around preserving functional vision for as long as possible and minimizing surgical procedures. Pharmacological treatment options aiming to reduce mutated TGFBIp levels in corneal cells in culture have been looked into by various researchers (Guo Sciriha et al. 2023) and topical formulations that aim to overcome the hurdle of penetrating the corneal physiological barrier are being explored (Cao et al. 2023; Baran-Rachwalska et al. 2020; SchiroLi et al. 2019; Irimia et al. 2018).

Genome editing has shown promise of being a game-changer for the management of rare hereditary genetic diseases, including previously untreatable ocular conditions (Yan et al. 2023). Conditions such as *TGFBI* CDs would theoretically being ideal candidates. Thus, the introduction of gene therapy procedures, with the aim of trying to prevent or stop the deposition of corneal material in these dystrophies as a non-surgical treatment option in CDs, is the way forward.

The two objectives of this study were thus as follows. Firstly, this study aimed to perform comprehensive transcriptome analysis using bioinformatics tools to investigate the differential expression between normal human corneal epithelial cells (HCECs) and *TGFBI* KD HCECs. The results would help to elucidate which genes are associated with the *TGFBI* gene, the effect inhibition of the *TGFBI* gene would have on downstream signaling pathways and thus on corneal structure and function, as well as imply which genes, other than TGFBI, might play a leading role in *TGFBI* CDs. It would also serve to delineate the role *TGFBI* plays in the homeostasis of corneal epithelial cells. The secondary objective of this study was to explore the possibility of gene therapy by exploring the effectiveness of shRNA in the inhibition of TGFBIp production. Theoretically, *TGFBI* KD in HCECs could by applied as a treatment modality for *TGFBI* CDs since it would prevent deposition of corneal complexes in the early stages of these conditions, keeping the cornea transparent.

## Methodology

The adult human corneal epithelium is the primary source of TGFBIp (MalkonDu et al. 2020; Niu et al. 2012) so, in order to explore the inhibition of *TGFBI* as a treatment modality, the first step involved establishing a cell culture of human corneal epithelial cells.

### Culture of Human Corneal Epithelial Cells

Human Corneal Epithelial cells (HCEC) (SCCE016, Sigma-Aldrich) and EpiGRO Human Ocular Epithelia Complete Media Kit were purchased from Merck Millipore (Sigma-Aldrich). The HCECs were cultured in EpiGRO complete medium in a 5% CO2 incubator at 37 °C. When the cells reached 80% confluency trypsin-EDTA digestion solution was added to detach cells from tissue culture dishes and to dissociate cells from one another. The suspension was subcultured for 3 more generations. HCECs were used in this project to address two issues in which there is a research void to date. The first was to seek a potential method of inhibiting TGFBIp production in the human cornea by exploring the effectiveness of shRNA in the inhibition of TGFBIp production. The second objective was to investigate, by means of bioinformatics tools, the effect the inhibition of *TGFBI* would have on downstream signaling and subsequently on corneal epithelial function.

### Induction of TGFBI Knockdown in Human Corneal Epithelial Cells

The induction of *TGFBI* knockdown in HCECs using a lentiviral vector containing shRNA sequences targeting the *TGFBI* gene was planned and subsequently conducted by LabOmics laboratories. The minimum antibiotic concentration required to kill 100% of cells in 72 hours was determined. This was found to be 2 µg/mL Puromycin. The vector used in this study was pHBLV-U6-MCS-CMV-ZsGreen-PGK-PURO. In order to construct the h-*TGFBI* shRNA Lentiviral Vector, first, the primers for the target fragment were selected. This was followed by the designing and synthesis of the shRNA oligonucleotides. The vector was digested with appropriate restriction enzymes a linearized vector was obtained by using a gel extraction kit. The target fragment was inserted into the linearized vector using T4 ligase. Competent DH5*α* cells were transformed and incubated for 12-16 hrs. Single colonies for sequencing validation were then selected and the plasmid was extracted from validated clones.

When designing the shRNA oligonucleotides to be used in this study, general guidelines that have been documented in literature, were followed (Li et al. 2007; Brummelkamp et al. 2002). Furthermore, target sequences were tested for homology using the BLAST function in NCBI database (NCBI 2016). Additionally, a control shRNA sequence was also designed to ensure the validity of the study (Jackson and Linsley 2004) (Table 1 and 2). In order to produce high-titer lentiviral particles, HEK293 cells were co-transfected with the lentiviral vector carrying the shRNA, a packaging vector (psPAX2) and an envelope vector (pMD2G). Transduction of the HCECs with HBLV-h-*TGFBI* -shRNA1-ZsGreen-PURO, HBLV-h-*TGFBI*-shRNA2-ZsGreen-PURO, HBLV-h-*TGFBI*-shRNA3-ZsGreen-PURO, HBLV-ZsGreen-PURO negative control, and HBLV-ZsGreen-PURO scrambled control viruses was performed, followed by antibiotic selection of positive knockdown cells.

**Table 1:** The control viral vector siRNA and shRNA sequence used in this study are:

Name	Sense/Antisense	Sequence
Control siRNA		TTCTCCGAACGTGTCACGTAA
Control shRNA	Sense	GATCCGTTCTCCGAACGTGTCACGTAATTCAAGAGATTACGTGACACGTTCGGAGAATTTTTTC
	Antisense	AATTGAAAAAATTCTCCGAACGTGTCACGTAATCTCTTGAATTACGTGACACGTTCGGAGAACG

**Table 2:** The target viral vector seq﻿uences and t﻿he three shRNA sequences used in this study are:

Name	Sense/Antisense	Sequence
siRNA1		CACCACTATCCTAATGGGATTGTAA
shRNA1	Sense	5’-GATCC**GCACCACTATCCTAATGGGATTGTAA**TTCAAGAGA**TTACAA TCCCATTAGGATAGTGGTG**TTTTTTG-3’
	Antisense	5’-AATTCAAAAAA**CACCACTATCCTAATGGGATTGTAA**TCTCTTGAA**TTA CAATCCCATTAGGATAGTGGTGC**G-3’
siRNA2		TCCACTACATTGATGAGCTACTCAT
shRNA2	Sense	5’-GATCC**GTCCACTACATTGATGAGCTACTCAT**TTCAAGAGA**ATGAGT AGCTCATCAATGTAGTGGA**TTTTTTG-3’
	Antisense	5’-AATTCAAAAAA**TCCACTACATTGATGAGCTACTCAT**TCTCTTGAA**ATG AGTAGCTCATCAATGTAGTGGAC**G-3’
siRNA3		CACATTGGTGATGAAATCCTGGTTA
shRNA3	Sense	5’-GATCC**GCACATTGGTGATGAAATCCTGGTTA**TTCAAGAGA**TAACAGG ATTTCATCACCAATGTG**TTTTTTG-3’
	Antisense	5’-AATTCAAAAAA**CACATTGGTGATGAAATCCTGGTTA**TCTCTTGAA**TAA CCAGGATTTCATCACCAATGTGC**G-3’

Bold values are the target sequence and its complimentary sequence. The middle sequence between these is the short loop that links them together

### Calculation of TGFBI Knockdown Efficiency Using qPCR

*TGFBI*-shRNA and non-silencing shRNA-transfected cells were harvested after 48-hour incubation. The RNA was subsequently purified using RNA purification kit. The purified RNA was reverse-transcribed into cDNA, transferred to a separate tube and used as template for qPCR. QPCR was performed on a Real-Time PCR Detection System using gene-specific primers for either *TGFBI* or GAPDH. The primers used for *TGFBI* and *GAPDH* are shown in Table 3 (Thornton and Basu 2015):

**Table 3:** Primers used in qPCR

Primers used for *TGFBI*		
	Length	GC%
Sense: GTGCTGTGCAGAAGGTTATTG	21	47.6
Antisense: CGTAGCTGATGACTGTTGATTTG	23	43.5
Primers used for ***GAPDH***		
Sense: GCCAAAAGGGTCATCATCTC	20	50%
Antisense: GGTGCTAAGCAGTTGGTGGT	20	55%

The percentage of siRNA-induced knockdown of *TGFBI* gene in the human corneal epithelial cells was calculated using the comparative CT method (∆∆CT) by applying the following formula:

∆ CT (sample) = CT target gene – CT reference gene

∆CT (calibrator) = CT target gene – CT reference gene

∆CT = ∆CT (sample) – ∆CT (calibrator)

Normalized target gene expression in sample = 2-∆∆CT

Knockdown efficiency (%) = (1—normalized target gene expression in sample) × 100%

Where: sample = the *TGFBI* -specific–siRNA transfection; calibrator = the non-silencing-siRNA transfection

### RNA Extraction and RNA Sequencing

RNA extraction was performed on the five control HCECs (3 negative controls and 2 scrambled controls) and the three shRNA *TGFBI* KD HCECs (shRNA1, 2, and 3) using the ReliaPrep™ RNA Miniprep System (Promega). Consequently, RNA sequencing libraries were prepared using TruSeq Stranded mRNA library kit (llumina 2018) according to manufacturer’s instructions and sequencing was performed on the Illumina HiSeq2500 sequencer at Dante Labs, Italy. This provided us with a snapshot of the cells’ pool of RNAs that could be subsequently used for differential expression analysis between the samples.

### Transcriptome Analysis to Investigate the Differential Expression of Genes Between Normal HCECs and TGFBI KD HCECs

RNA-seq analysis was performed on a short read dataset that was obtained using Illumina next generation sequencing technology. Pre-processing of the raw files was performed using Trimmometic and the alignment of the clean RNA-seq reads (FastQ file) to the human reference genome GRCh37 was performed using the HISAT2 software. SAM files were converted to BAM format, sorted and then indexed, in order to obtain the analysis-ready reads. In order to generate counts of reads mapped to known genes, the alignment files were provided as input to *featureCounts* for the quantification step. The counts were summarized and reported using the *geneId* feature in the annotation file and were saved to count files, which serve as input for downstream RNA-seq differential expression analysis (Summary of counting results can be found in the “RAW counts matrix.csv” file in the github repository).

The pyDESeq package, which is a python implementation of DESeq2, was used to perform differential expression analysis of genes between *TGFBI* KD HCECs (included shRNA1, shRNA2, and shRNA3) and normal HCECs (included all five controls). The RNA-seq raw count data were used as an input for the pyDESeq package (The code compiled in order to compare the KD with the control can be found in the appendix/github repository). The main output result of the differential expression analysis carried out is a list of differentially expressed (DE) genes. Further filtering produced a list with the most significant overlapping over and under expressed genes from shRNA1, shRNA2, and shRNA3. Only these DE genes were taken into consideration in the enrichment analysis in order to strengthen the confidence that the changes identified were due to the *TGFBI* KD. (This list can be found in the appendix/github repository).

### Enrichment Analysis

Bioinformatics were used to perform functional enrichment analysis by delineating critical pathways, via KEGG and Reactome databases, and to identify molecular and biological functional GO annotations of the differentially expressed genes. The web-based GEne SeT AnaLysis Toolkit (WebGesalt) was employed to perform an Over-Representation Analysis (ORA) in function and pathways. The parameters used to run ORA included the following: Organism of Interest: Homo sapiens; Method of interest: Over-Representation Analysis; Functional Database: Gene Ontology- Biological process, Molecular function or Pathway- KEGG, Reactome for every set of DE genes; Gene ID type inserted was the Gene symbol; Gene lists uploaded were the up or downregulated DE expressed genes for comparison group; and Reference set chosen: genome. The advanced parameters included Minimum number of genes for a category: 5; Maximum number of genes for a category: 2000; Multiple test adjustment: BH; Significance level: top 25; Number of categories expected from set cover: 10; and Number of categories visualized in the report: 40. The significance threshold applied was ≤ 0.05 for each group of enriched genes.

The genes that showed the largest log2FCs were looked into. Additionally this study also honed in on and identified the GO annotations and related pathways of ‘genes of interest’ known to be related to *TGFBI*, which were shortlisted from literature and databases as well as proteins that have been shown to be present in *TGFBI* CD deposits. The ‘genes of interest’ analyzed in this study were: *A2M, COL2A1, COL4A1, COL4A2, COL4A3, COL4A4, COL6A5, CPA2, CPA4, EMILIN3, ESR1, ESR2, FLOT1, FLOT2, FN1, HSPG2, ITGA3, ITGA5, ITGA11, ITGAM, ITGAV, LOXL2, ITGB5, MAPK1, MAPK3, MMP2, NOG, PLOD2, POSTN, RAP2B, RELA, SELE, STAB1, STAB2, S100P, THBS1, TNC, TNF, VCAM1, ZC2HC1A, AKT1, PIK3CA (PI3K), GSK3, CUL4A, IL1B, IL6, JAK2, STAT3, SMAD2, SMAD3, SMAD4, SMAD7, TGFBI, COL1A1, COL1A2, HBB, LUM, DCD, KERA, COL6A3, ALDH3A1, COL6A1, COL6A2, DCN, ALB, ENO1, COL3A1, COL5A1, ACTG1, COL5A2, CLU, BGN, LDHA, MAMDC2, COL12A1, VIM, APCS, CSTA, S100A9, S100A6, S100A4, APOD, APOA4, ANXA2, IGKC, IGHG3, IGHG1, H2B A1, RPS27a, LYZ, PTGDS, PRDX1, FABP5, HSPB1*.

This comprehensive transcriptome and enrichment analysis helps to give insight into the significance these DE genes have with regards to their association with *TGFBI*, together with the potential response to future treatment options.

## Results

### Sequencing Validation of Positive Clones

**Fig. 1:**
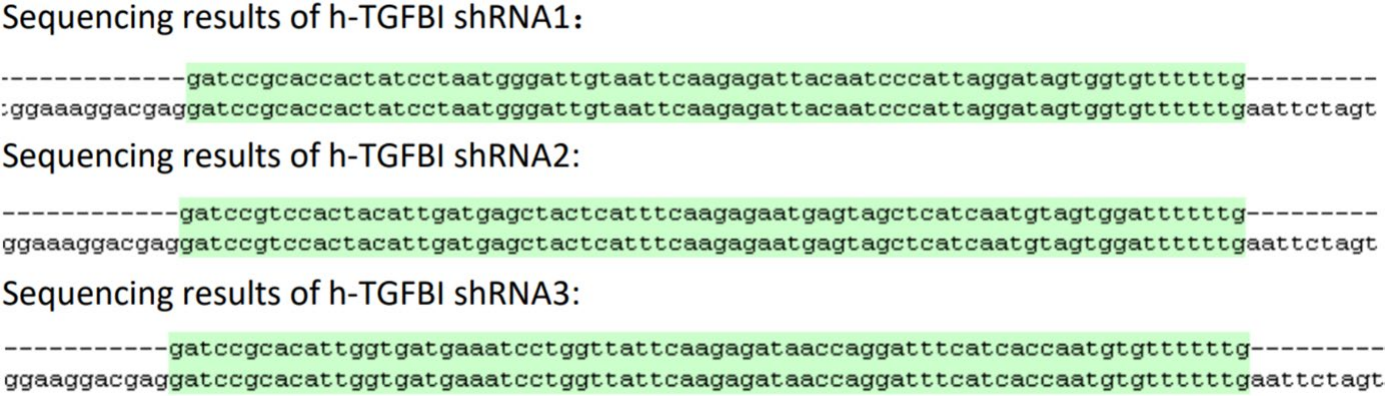
Sequencing results from positive clones of h-TGFBI shRNA1, 2, and 3

A single colony from each plate was picked and positive clones were identified by sequencing (Fig. 1).

### KD Efficiency

**Fig. 2:**
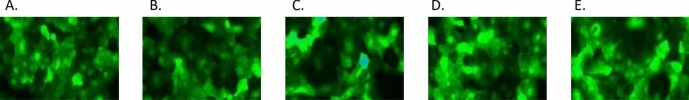
Green fluorescence protein expression in human corneal epithelial cells at day 3 post transduction. The above are fluorescence images of hCECs transduced with (A) scrambled control, (B) empty vector control, (C) HBLV-h-*TGFBI* shRNA1-ZsGreen-PURO, (D) HBLV-h-*TGFBI* shRNA2-ZsGreen-PURO, and (E) HBLV-h-*TGFBI* shRNA3- ZsGreen-PURO

The transduction check was performed by observing under a fluorescence microscope the HCECs transduced with the scrambled shRNA, empty vector, HBLV-h-*TGFBI* shRNA1-ZsGreen-PURO, HBLV- h-*TGFBI* shRNA2-ZsGreen-PURO, and HBLV- h-*TGFBI* shRNA3- ZsGreen-PURO (Fig. 2).

### Positive KD Check

**Fig. 3:**
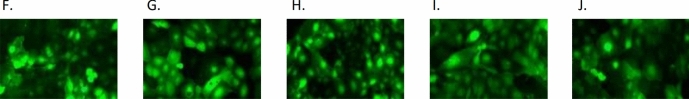
Lentivirus knockdown efficiency recorded under a fluorescence microscope: (F) scrambled control, (G) empty vector control, (H) HBLV-h-*TGFBI* shRNA1-ZsGreen-PURO, (I) HBLV-h-*TGFBI* shRNA2-ZsGreen-PURO, and (J) HBLV-h-*TGFBI* shRNA3- ZsGreen-PURO

Lentivirus knockdown efficiency was checked under a fluorescence microscope (Fig. 3).

### TGFBI KD Efficiency Using qPCR

KD efficiency was determined at the RNA level through both RT-qPCR and RNAseq, with results demonstrating a 70.5% (shRNA1) reduction in TGFBI transcript levels by qPCR (Table 4) and up to 88% (shRNA2) by RNAseq data analysis. These findings are supported by detailed RT-qPCR methodology and fluorescence validation conducted by our collaborators at Labomics S.A., provided as supplementary documentation (KD Determination Report and KD Cell Pools Development Report).

**Table 4:** Threshold Cycle (CT) Values

	TGFBI specific PCR primer, 3 replicates	Average TGFBI specific PCR primers	GADPH-specific PCR primers, 3 replicates	Average GADPH-specific PCR primers
Transfected with TGFBI siRNA1	26.20; 27.33; 28.47	27.33	15.25; 15.37;15.81	15.48
Transfected with non-silencing siRNA (NC)	25.56; 25.37; 25.35	25.43	15.31; 15.46; 15.24	15.34

The efficiency of shRNA1 was calculated to be 70.5% using the comparative CT method and 73% by calculating the efficiency from RNA-seq normalized values. The efficiency of shRNA2 and shRNA3 were calculated to be 88% and 55%, respectively, from analysis of RNA-seq data.

### Differential Expression Analysis

**Fig. 4:**
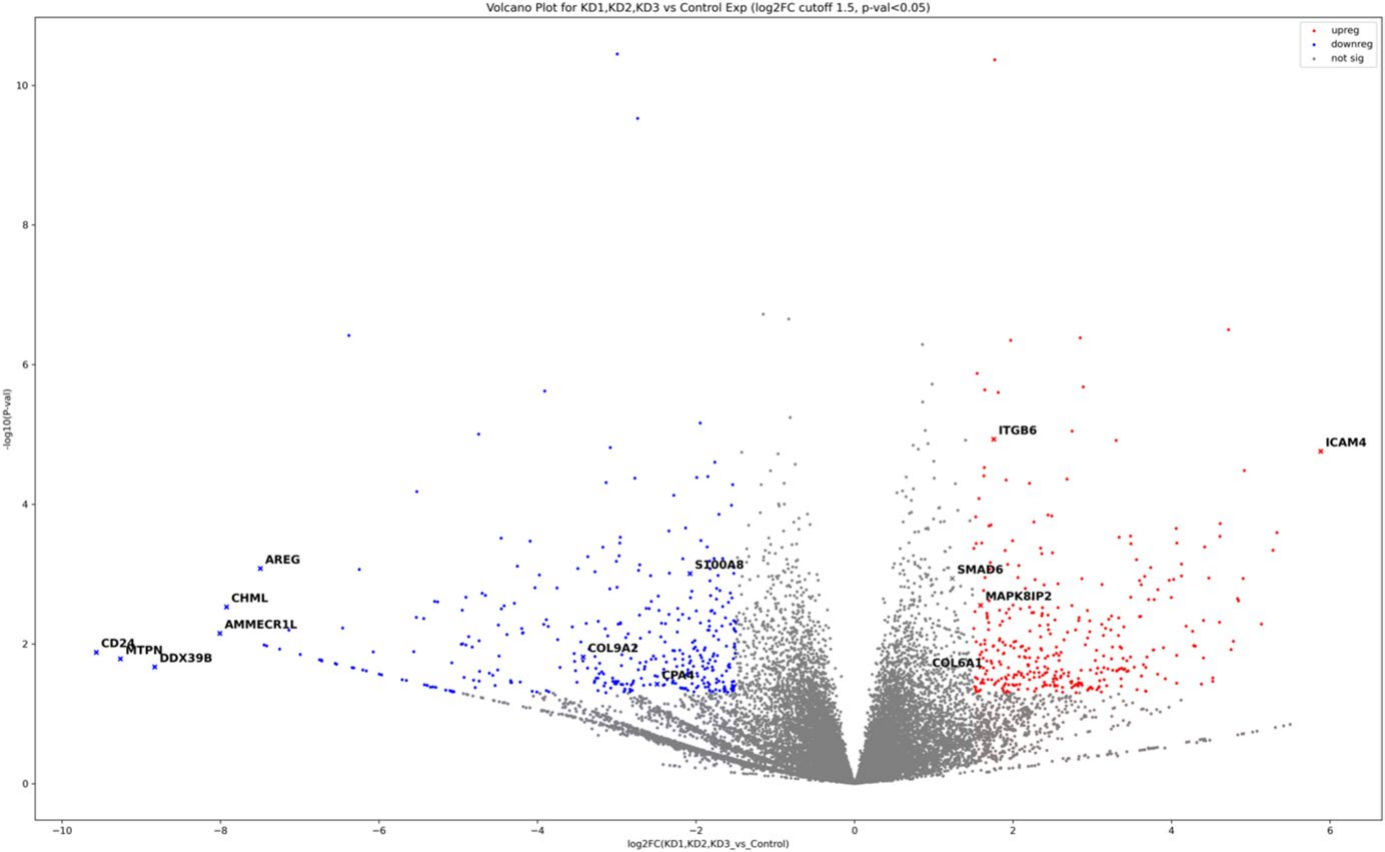
Volcano plot representation of DE genes from differential expression analysis of the RNAseq data obtained in the *TGFBI* KD HCEC group vs Control group. The figure indicates up-regulated and down-regulated genes when comparing the *TGFBI* KD HCEC group with the control group

The total number of DE genes found in *TGFBI* KD HCECs was 2296, with a log2FC cutoff of 1.5 decreasing the number to 742. A volcano plot displaying the DE genes from differential expression analysis of the RNAseq data obtained in the *TGFBI* KD HCEC group vs Control group is shown in Figure 4. The number of up and downregulated DE expressed genes can be found in Table 5.

**Table 5:** Number of total up and downregulated DE expressed genes (P ≤ 0.05) and number of DE genes with cut off of 1.5 log2FC for *TGFBI* KD HCECs vs Control HCECs

	TGFBI KD HCECs
Total Downregulated DE genes	1180
Downregulated DE genes with log_2_FC ≤ −1.5	365
Total Upregulated DE genes	1116
Upregulated DE genes with log_2_FC ≥ 1.5	377

A total of 1180 downregulated DE genes in KD HCECs with p value of ≤0.05 were identified by DE analysis. When a log2FC cut-off of ≤−1.5 was implemented, the number of significantly downregulated genes went down to 365. When eliminating pseudogenes/unclassified GO annotated genes, this number was further reduced to about 152 genes. The downregulated genes that showed the largest log2FCs were looked into (Table 6). Furthermore, this study also honed in on and analyzed the ‘genes of interest’ known to be related to *TGFBI* (Table 7).

**Table 6:** Downregulated genes in *TGFBI* KD HCECs that showed the largest log_2_FCs

Genes with largest log_2_FC Downregulated in KD	Name	Log_2_FoldChange	P value
*CD24*	Cluster of differentiation 24	− 9.56923	0.01317
*MTPN*	Myotrophin	− 9.26364	0.016398
*DDX39B*	DEAD box helicase 39b	− 8.83188	0.021397
*AMMECR1L*	AMME Chromosomal Region Gene 1-Like	− 8.01021	0.007045
*CHML*	CHM Like Rab Escort Protein	− 7.92606	0.002959
*AREG*	Amphiregulin	− 7.49931	0.000831

**Table 7:** Genes of interest that were found to be downregulated in *TGFBI* KD HCECs

Genes of interest Downregulated in KD	Name	Log_2_FoldChange	P value
*CPA4*	Carboxypeptidase A4	− 2.49358	0.037592
*IL6*	Interleukin 6	− 1.53083	0.046589
*ITGA11*	Integrin subunit alpha-11	− 1.34462	0.042883
*S100A9*	S100 calcium-binding protein A9	− 0.93364	0.04691
*IL1B*	Interleukin 1B	− 0.90163	0.01318
*JAK2*	Janus Kinase 2	− 0.68498	0.020046
*STAT2*	Signal transducer and activator of transcription 2	− 0.59388	0.000137

A total of 1116 upregulated DE genes in KD HCECs with p value of ≤0.05 were identified by DE analysis. When a log2FC cut-off of ≥1.5 was implemented, the number of significantly upregulated genes went down to 377. Eliminating pseudogenes/unclassified GO annotated genes reduced the number further to 140 genes. The upregulated genes that showed the largest log2FCs were looked into (Table 8). Furthermore, similarly to what was done with the upregulated genes, the downregulated ‘genes of interest’ known to be related to *TGFBI* (Table 9), which were shortlisted from literature and databases, as well as proteins that have been shown to be present in *TGFBI* CD deposits, were analyzed.

**Table 8:** Upregulated genes in *TGFBI* KD HCECs that showed the largest log2FCs

Genes with largest log_2_FC Upregulated in KD	Name	Log_2_FoldChange	P value
*ICAM4*	Intercellular adhesion molecule 4	5.881498	1.75E-05
*RNA5SP439*	RNA, 5S Ribosomal Pseudogene 439	5.330029	0.000255
*RNA5S17*	RNA, 5S Ribosomal 17	5.27924	0.000454
*SNORD14C*	Small Nucleolar RNA, C/D Box 14C	5.133837	0.005183
*RN7SL385P*	RNA, 7SL, Cytoplasmic 385	4.918127	3.28E-05
*ANKRD20A10P*	Ankyrin repeat domain 20	4.902211	0.001153

**Table 9:** Genes of interest that were found to be upregulated in *TGFBI* KD HCECs

Genes of interest Upregulated in KD	Name	Log_2_FoldChange	P value
*MMP2*	Matrix metalloproteinase 2	1.253716544	0.022905
*SMAD6*	Suppressor of Mothers against Decapentaplegic 6	1.23267	0.001155
*COL6A2*	Collagen Type VI Alpha 2 Chain	0.999639247	0.023426
*COL6A1*	Collagen Type VI Alpha 1 Chain	0.916271641	0.025146
*AKT1*	Ak strain transforming	0.583362324	0.002419
*ITGA6*	Integrin subunit alpha 6	0.353225934	0.02616

### Enrichment Analysis

**Fig. 5:**
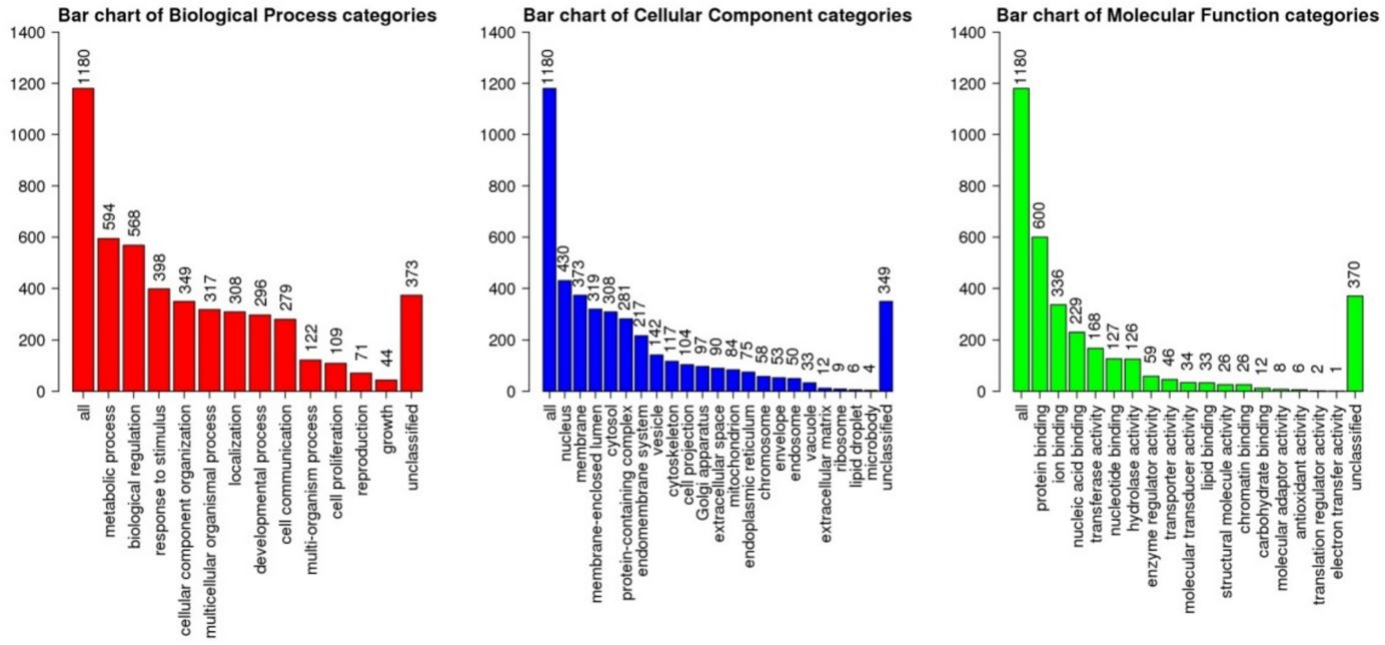
Bar charts displaying enrichment results of GO annotations of downregulated genes identified from differential expression analysis of the RNAseq data in KD vs Control groups

**Fig. 6:**
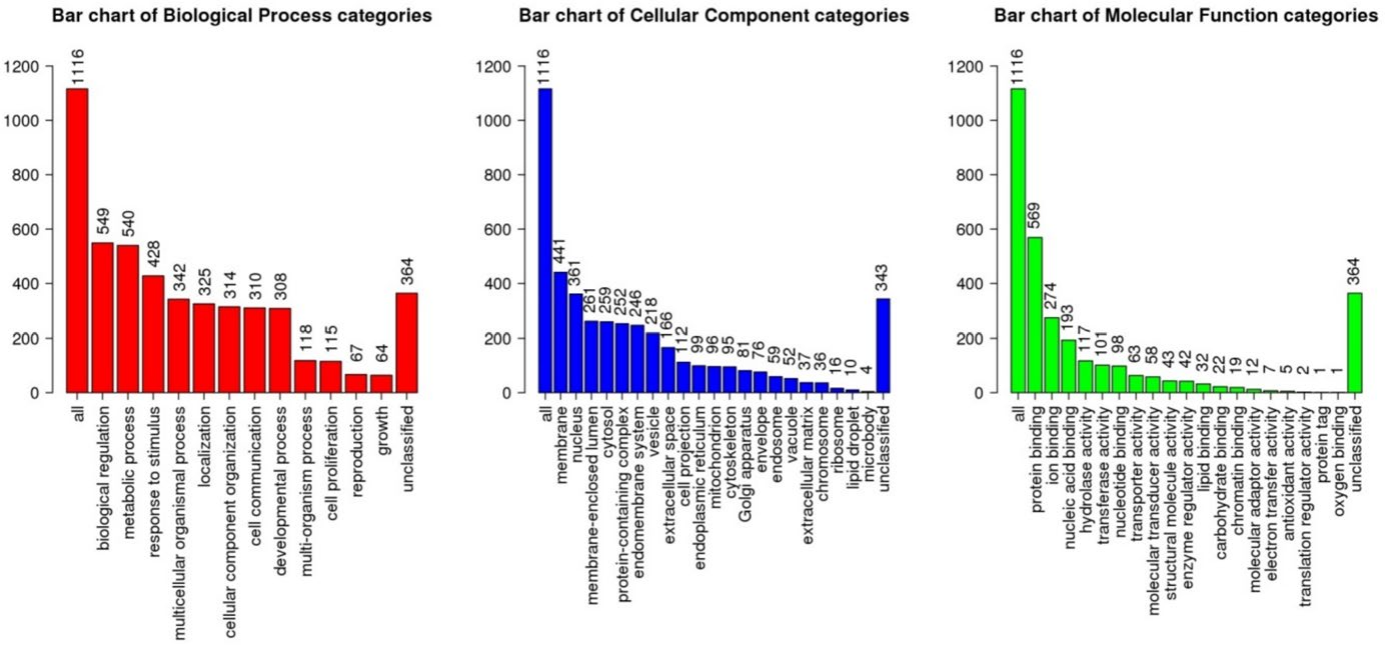
Bar charts displaying enrichment results of GO annotations of upregulated genes identified from differential expression analysis of the RNAseq data in KD vs Control groups

A summary of the results obtained from ORA analysis for upregulated and downregulated DE genes identified in *TGFBI* KD HCECs vs Control HCECs can be found in Figures 5 and 6 and Tables 10 and 11, where the first 25 descriptions (P ≤0.05) for each set are listed (the full set of results including the GO annotation or pathway, gene overlap, p value and gene IDs for each of the described processes can be found in the appendix/github repository).

**Table 10:** GO annotations and pathway associations of downregulated genes identified from differential expression analysis of the RNAseq data in KD vs Control groups

GO Biological Process	GO Molecular Function	KEGG Pathway	Reactome Pathway
Cellular response to stress	Phosphotransferase activity, alcohol group as acceptor	Basal transcription factors	Regulation of TP53 Activity
Cellular response to external stimulus	Histone demethylase activity	Autophagy/Mitophagy/Endocytosis	RNA Polymerase II Transcription
Cellular response to DNA damage; DNA repair	Kinase activity	RNA transport	Metabolism of RNA
Cell cycle	ATP binding	p53 signaling pathway	Transcriptional activity of SMAD2/SMAD3:SMAD4 heterotrimer
Regulation of signal transduction by p53 class mediator	Modification-dependent protein binding	Phosphatidylinositol signaling system	Phosphatidylinositol Metabolism/Synthesis of PIPs at the plasma membrane
SnRNA transcription by RNA polymerase II	Purine ribonucleoside triphosphate binding	FoxO signaling pathway	Phospholipid metabolism
Regulation of cellular component biogenesis	Polyubiquitin modification-dependent protein binding	NOD-like receptor signaling pathway	Homology-Directed-Repair through Single Strand Annealing (SSA) and Homologous Recombination (HRR)
Protein dealkylation	Phosphatidylinositol bisphosphate phosphatase activity	NF-kappa B signaling pathway	Presynaptic phase of homologous DNA pairing and strand exchange
Protein and Histone lysine demethylation	Protein serine/threonine kinase activity	TNF signaling pathway	Interleukin-20 family signaling
Non-motile cilium assembly	Mannosidase activity	mTOR signaling pathway	Homologous DNA Pairing and Strand Exchange
Peptidyl-serine phosphorylation	Carbon–sulfur lyase activity	IL-17 signaling pathway	SUMOylation of DNA damage response and repair proteins
Organelle assembly	K63-linked polyubiquitin modification-dependent protein binding	Cell cycle	Negative regulators of DDX58/IFIH1 signaling
Golgi organization	Drug binding	Fanconi anemia pathway	Fanconi Anemia Pathway
		Amino sugar and nucleotide sugar metabolism	Infectious disease/HIV life cycle

**Table 11:** GO annotations and pathway associations of upregulated genes identified from differential expression analysis of the RNAseq data in KD vs Control groups

GO Biological Process	GO Molecular Function	KEGG Pathway	Reactome Pathway
Regulation of apoptotic signaling pathway	Transmembrane transporter activity (proton and nucleoside)	Alzheimer, Huntington, Parkinson disease	Apoptosis
Cell activation/Cellular response to external stimulus and Positive regulation of signaling	Transferase activity, transferring acyl groups]/Lysophosphatidic acid acyltransferase activity	Axon guidance	The citric acid (TCA) cycle and respiratory electron transport
Mitochondrial membrane organization/Apoptotic mitochondrial changes	Sequence-specific double-stranded DNA binding	Oxidative phosphorylation	Transport of nucleosides and free purine and pyrimidine bases across the plasma membrane
Regulation of protein localization to membrane	Identical protein and Tau protein binding	Thermogenesis	Metabolism of nitric oxide
Positive regulation of interleukin-8 production	MHC class II protein complex binding	Glucagon signaling pathway	eNOS activation and regulation
Exocytosis	Transcription regulatory region sequence-specific DNA polymerase binding/RNA polymerase II-specific	Vasopressin-regulated water reabsorption/Renin secretion	Tetrahydrobiopterin (BH4) synthesis, recycling, salvage, and regulation
Activation of cysteine-type endopeptidase activity	Carbohydrate binding	Metabolic pathways (Glutathione metabolism)	Nef Mediated CD4 Down-regulation
Regulation of binding	Transcription coactivator activity	Human papillomavirus infection	Respiratory electron transport, ATP synthesis
Neutrophil mediated immunity	Myosin binding	Folate biosynthesis	Interleukin signaling
Secretion		Gap junction	Detoxification of ROS
Vesicle-mediated transport		ECM-receptor interaction	Neutrophil degranulation
		Glycosphingolipid biosynthesis	Translocation of SLC2A4 (GLUT4) to the plasma membrane
		Focal adhesion	Metabolism of cofactors
		Cortisol synthesis and secretion	Neurodegenerative Diseases

## Discussion

### The Elusive Physiological Role of TGFBIp

*TGFBI* expression in corneal epithelial cells can be induced by TGF-*β*1 (Guo et al. 2017; Yellore et al. 2011; Wang et al. 2002). The binding of TGF-*β* to its receptor can theoretically lead to the activation of either the SMAD signaling pathway or else non-SMAD signaling pathways that include: PI3K/AKT, MAPK pathways (ERK, JNK, and p38 MAPK) as well as NF-*κ*B and Rho/Rac1 pathways, among others. In fact, besides the direct regulation by the TGF-*β*/SMAD signaling pathway, the expression of the *TGFBI* gene is also modulated indirectly by the PI3-K/AKT, the cAMP/PKA, and the JNK signaling cascades (Guo et al. 2017).

However, critical questions persist regarding TGFBI’s cell-type-specific mechanisms controlling its induction and what regulatory factors influence its expression. This gap is particularly striking given TGFBIp’s abundance in the human cornea, with its physiological role continuing to elude definitive characterization (CHen et al. 2020). In fact, a recent study carried out by (Nielsen et al. 2020) further highlighted the necessity of research focusing on the function of TGFBIp in the cornea, which is one of the objectives of this study.

### Downregulated Differentially Expressed Genes in TGFBI KD HCECs

The pattern of the downregulated DE genes suggests multiple interconnected biological effects.

The *CD24* gene encodes a sialoglycoprotein involved in granulocytes and B cell signaling, angiogenesis (via Hsp90-mediated STAT3/VEGF), and it is stimulated by TGF-*β* (Nakamura et al. 2017). *DDX39B*, an RNA helicase, plays a role in mRNA export from the nucleus to the cytoplasm, including export of FUT3, which fucosylates TGF-*β*R-I, activating the TGF-*β* signaling pathway (He et al. 2021). This could be a reflection of the mechanism brought about by the shRNA itself.

The protein encoded by the *AREG* gene, amphiregulin, is a transmembrane glycoprotein related to epidermal growth factor (EGF) and transforming growth factor alpha (TGF-*α*). It promotes epithelial cell proliferation via the EGF receptor. Generally, TGF-*α* is known to promote cell proliferation while the versatile TGF-*β* may stimulate or inhibit proliferation depending on the type of cells and growth factors involved (Chia et al. 2024). (e.g., AREG activates the EGFR/JNK/AP-1 pathway that mediates the TGF-*β*- induced epithelial mesenchymal transition in lung cells) (CHeng et al. 2022).

The gene *S100A9* codes for a calcium- and zinc-binding protein that modulates inflammatory and immune responses. It has also been implicated in the pro-inflammatory IL-17 signaling pathway (that activates NF-kappaB and MAPK pathways) identified during KEGG pathway analysis. Interestingly, (Courtney et al. 2015) reported the accumulation of S100A9 in LCD1 TGFBIp deposits, while usually this protein is not found to be present within the healthy cornea. Thus, downregulation of S100A9 seen in *TGFBI* knocked down corneal epithelial cells could additionally lead to decreased deposition of complexes if implemented as treatment in LCD1 individuals, providing further benefit.

IL6 is a cytokine that has been documented to have both pro- and anti-inflammatory properties (AliYu et al. 2022; ScHeller et al. 2011). Previous studies carried out on HK-2 cells (human renal proximal tubular epithelial cells immortalized by transduction with human papilloma virus 16 E6/E7 genes) have indicated that IL-6 increased trafficking of TGF-*β*1 receptors that in turn resulted in amplified TGF-*β*1 SMAD signaling (Elkoshi 2024; ZHang et al. 2005). In this study *TGFBI* KD resulted in downregulation of IL6 expression; this might imply that these two proteins are positively correlated when it comes to corneal healing and inflammatory response. IL6 is also known to activate JAK2 (Janus Kinase 2 gene)/STAT3thus modulating cellular proliferation and ‘cellular response to stress.’

Another downregulated gene found in *TGFBI* KD HCECs in this study was *IL1B,* a pro-inflammatory cytokine reported to cause persistent severe inflammation of the ocular surface (Liu et al. 2012). Interestingly, TGF-*β* and IL1 have been labeled as ‘master regulators’ of the corneal wound healing reaction to injury with reports of opposing but occasionally also supporting roles (Wilson 2021). Its related pathways include IL-17, TNF, and NF-kappa B.

The gene *ITGA11* encodes integrin alpha-11 protein, which is induced by TGF- *β*1 via SMAD2 in primary fibroblasts, thus being linked to corneal collagen deposition and scarring in keratoconic corneas (Bystrom et al. 2009). In the *TGFBI* KD HCECs in this study, *ITGA11* was found to be downregulated, thus inferring possibly reduced fibrosis.

On the other hand, the main role of STAT2 is to transduce signals downstream of the receptors for IFN-I and –III and modulates inflammation (Duncan and Hambleton 2021; KalLiolias and Ivashkiv 2010).

In summary, Webgestalt analysis indicated downregulated genes (p ≤ 0.05) in KD HCECs were related to the cell cycle (specifically p53 signaling), cellular response to stress and transferring of phosphorus-containing groups. Related pathways included IL-20, IL-17, TNF, NF-kappa B, and transcriptional activity of SMAD2/SMAD3:SMAD4 heterotrimer.

The function of the downregulated DE genes with a log2FC of ≤ −1.5 were analyzed in more detail and categorized according to physiological function (Table 12), COL8A2 and COL9A2, as well as *LOX*, the gene encoding for an enzyme that catalyzes the crosslinking of collagen and elastin fibers in the extracellular matrix, were also found to be downregulated. A subset of genes involved in RNA processing, were also enriched (*SNORD3B-2, SNORD3B-1, SNORD3C, RPP14, HNRNPH2, SRSF10, SNRPN, and TRMT13*). However these might be due to the effect of shRNA transduction.

**Table 12:** Categorization of downregulated DE genes with a log2FC of ≤ −1.5 according to physiological function based on current literature

Cell adhesion and signaling	*CD24, NEDD9, SH3GL2, ST3GAL6, ST6GALNAC5, MPPE1,* *PCDHGC3, PCDHGB5, PCDHGB6, PCDHGA9, PCDHGA10, PCDHGB7, IFT80, IZUMO1, CLDN16, COL8A2, S100A8, CTNND1, MEGF11, IGSF1, SGK3, GNGT1, GNB3, RGS9, ANXA8*
Immune response	*CD24, IL24, IL20, IL23A, IL6, IRAK3, S100A8*
Growth factor/Cell proliferation	*AREG, SH3GL2, CSNK1E(Wnt), BOP1*
Inflammation	*AREG, IL24, IL20, IL6, IRAK3, S100A8*
Healing and tissue repair	*AREG, IL20, RTEL1*
Neurotransmitter/neuronal system involvement	*DNAJC6, SH3GL2, PCDHGC3, PCDHGB5, PCDHGB6, PCDHGA9, PCDHGA10, PCDHGB7, IGSF1, GABRP, GNGT1, GNB3*
Transcriptional regulation	*GTF2IRD2B, DDX39B, DQX1, PNRC2, SCX, TRPS1*

Thus the general trend of downregulated genes observed in *TGFBI* KD HCECs points toward a decrease in inflammation via downregulation of *IL1B, IL6,* and *JAK2*; decreased angiogenesis via downregulation of *CD4, IL6,* and *JAK2*; decreased TGF-*β* signaling via downregulation of *DDX39B* and *IL6*, and decreased fibrinectin production and corneal scarring via downregulation of *AREG* and *ITGA11*. The downregulation of genes related to ECM and cell adhesion hint toward ECM remodeling suppression. These results suggest that KD leads to a less inflammatory, less fibrotic, and potentially less angiogenic cellular environment. These general outcomes that would be theoretically seen by the mentioned downregulated genes were subsequently analyzed in combination with the upregulated genes in *TGFBI* KD HCECs.

### Upregulated Differentially Expressed Genes in TGFBI KD HCECs

Most of the significantly upregulated genes that were found to have the largest log2FC are pseudogenes, which, though non-protein coding, they can be processed into short interfering RNAs that can regulate the expression of coding genes at transcriptional and post-transcriptional level (RanganatHan and Sivasankar 2014).

The upregulated *ICAM4* gene encodes the intercellular adhesion molecule 4, which has been shown to serve as a ligand for *α*−4/*β*−1 and alpha-V integrins. *TGFBI* is known to bind to alpha-V integrins with high affinity.

*SMAD6* is an inhibitory SMAD and is known to negatively regulate TGF-*β* type1 signaling. It has been shown to preferentially inhibit BMP-induced SMAD signaling (Miyazawa and Miyazono 2017). However, it has also been shown to impede the phosphorylation of SMAD2 and the subsequent heterodimerization with SMAD4 (Yakymovych et al. 2006; INamura et al. 1997). DEseq analysis of DE genes in *TGFBI* KD HCECs showed an upregulation of *SMAD6* thus exhibiting a negative correlation with *TGFBI* expression.

*AKT1* encodes AKT Serine/Threonine kinase1, which plays a role in the modulation cell proliferation, metabolism, and angiogenesis in both normal and malignant cells via the PI3K/AKT pathway. It is also a known oncogene. PI3K/AKT signaling pathway molecules are known to mediate crosstalk with the TGFb pathway that in turn regulates TGFBIp production (Garg et al. 2025; Yu et al. 2015). Similarly to *AKT1, ITGA6* has also been implicated in the ‘regulation of apoptotic signaling pathway,’ ‘focal adhesion,’ ‘ECM-receptor interaction,’ and ‘Pathways in cancer,’ thus functioning as a cell surface adhesion molecule binding ECM molecules of the laminin family (Stelzer et al. 2016).

In this study, *COL6A* and *COL6A2*, encoding for collagen VI proteins, were both found to be up- regulated, suggesting a compensatory mechanism for the decreased *TGFBI* expression. Collagens have been reported to interact with several ECM proteins and cell surface molecules such as integrin proteins. Besides its important structural role, collagen VI affects various cellular functions including adhesion, migration, and cytoprotection (Cescon et al. 2015). Overexpression of COL6, observed in corneal wound healing (Esquenazi et al. 2009) and also in TGFBI-null mice (Poulsen et al. 2018), suggests that the covalent binding of TGFBIp to COL6 must be one of the important physiological roles of TGFBIp, assisting subsequent regulation of matrix organization and collagen constituent composition without producing significant structural disruption (Poulsen et al. 2018).

The gene *MMP2* that encodes the enzyme matrix metallopeptidase 2 was also found to be upregulated. It is known to play an important role in extracellular-remodeling during corneal wound healing (Li et al. 2001) and is positively associated with increased corneal fibrosis and neovascularization in corneal disease (Wolf et al. 2019).

Thus, the enriched pathways and GO annotations associated with all the significantly (p ≤0.05) upregulated DE genes in KD HCECs were mainly associated with cell cycle regulation (including the apoptotic signaling pathway), cell communication and signaling, ECM-receptor interaction, focal adhesion and identical protein binding. ‘Transcription’ and ‘RNA polymerase II regulatory region sequence-specific DNA binding’ annotations were also noted. However similarly to what was noted in the downregulated genes, these might be related to the method employed to induce the TGFBI KD in the HCECs, namely shRNA.

The function of the upregulated DE genes with a log2FC of ≥ 1.5 were looked into in more detail and categorized according to their physiological function (Table 13).

**Table 13:** Categorization of upregulated DE genes with a log2FC of ≥ 1.5 according to physiological function based on current literature

Cell adhesion and signaling	*MUC1, MAPK8IP2(JNK), LRP1, IFNAR2, ADCY8, VSTM2L, ITGB6, L1CAM, LY6D, CADM2, SELP, WNT10A, PDE1A, LRRC4B, ADCY8*
Immune response	*CFD, IL17D, IRF4, PRG2, CLEC4A, IL13, IFNL1, KLRC3, KLRC1EGR3, LY6D, LCK, CLEC4A, IL34, FCGR1A, CFI, CFH, SELP, IFNAR2*
Growth factor/Cell proliferation	*PDGFRB, FGF3*
Inflammation	*CFD, IL17D, MMP12, PRG2, IL13, SELP*
Healing and tissue repair	*FGF3*
Neurotransmitter/neuronal system involvement	*PDXP, NPY4R, NPFFR1, NTSR1, GABRQ, KCNC3, CPLX1, LRRC4B, KCNJ6, HTR3E, ADCY8*
Transcriptional regulation	*IRF4, CITED1, SOX8, EGR3, CNTN1, L1CAM, CADM2, CNTN5*

Overall, *TGFBI* KD in HCECs appears to inhibit TGF-β/SMAD and BMP signaling (*SMAD6* upregulation), promote ECM remodeling (*MMP2*, COL6), enhance proliferation (*AKT1*, *ITGA6*), and alter integrin signaling (*ICAM4*). The upregulation of collagen 6 suggests compensatory ECM restructuring in the absence of TGFBIp while the upregulation of genes involved in integrin signaling hint at reprogrammed cell-matrix interactions. However, concurrent downregulation of pro-inflammatory and pro-angiogenic genes (*IL6*, *JAK2*, *ITGA11*) may balance these effects, possibly reflecting compensatory mechanisms and highlighting the plasticity of corneal epithelial cells, in the attempt to maintain function despite *TGFBI* loss. Thus, these DE genes suggest that corneal epithelial cells adapt to *TGFBI* loss by reducing inflammation, modulating integrin-dependent adhesion signals and restructuring ECM via alternative collagen pathways.

From this analysis, corneal *TGFBI* appears to be critical in the maintenance of corneal integrity by promoting TGF-β/SMAD signaling, thus modulating inflammatory and fibrotic responses; in stabilizing the ECM by preventing excessive remodeling; and by modulating collagen and integrin interactions, thus regulating cell-matrix adhesion.

### TGFBI KD in HCECs and its Potential Implications for Understanding CDs

*TGFBI* CDs are a complex group of conditions in which various *TGFBI* mutations disrupt protein folding, stability, and ECM interactions leading to alterations in the overall role of the protein. These changes likely lead to dysregulation of *TGFBI*-associated genes.

The pathways leading to complex formation and deposition in *TGFBI* CDs, as well as the reason why the pathological manifestation of *TGFBI* mutations is confined only to the cornea, have yet to be uncovered.

KD experiments provide a controlled and precise way to dissect *TGFBI*’s role in the disease process, offering valuable insights that may not emerge from direct comparisons between healthy and diseased cells.

**Fig. 7:**
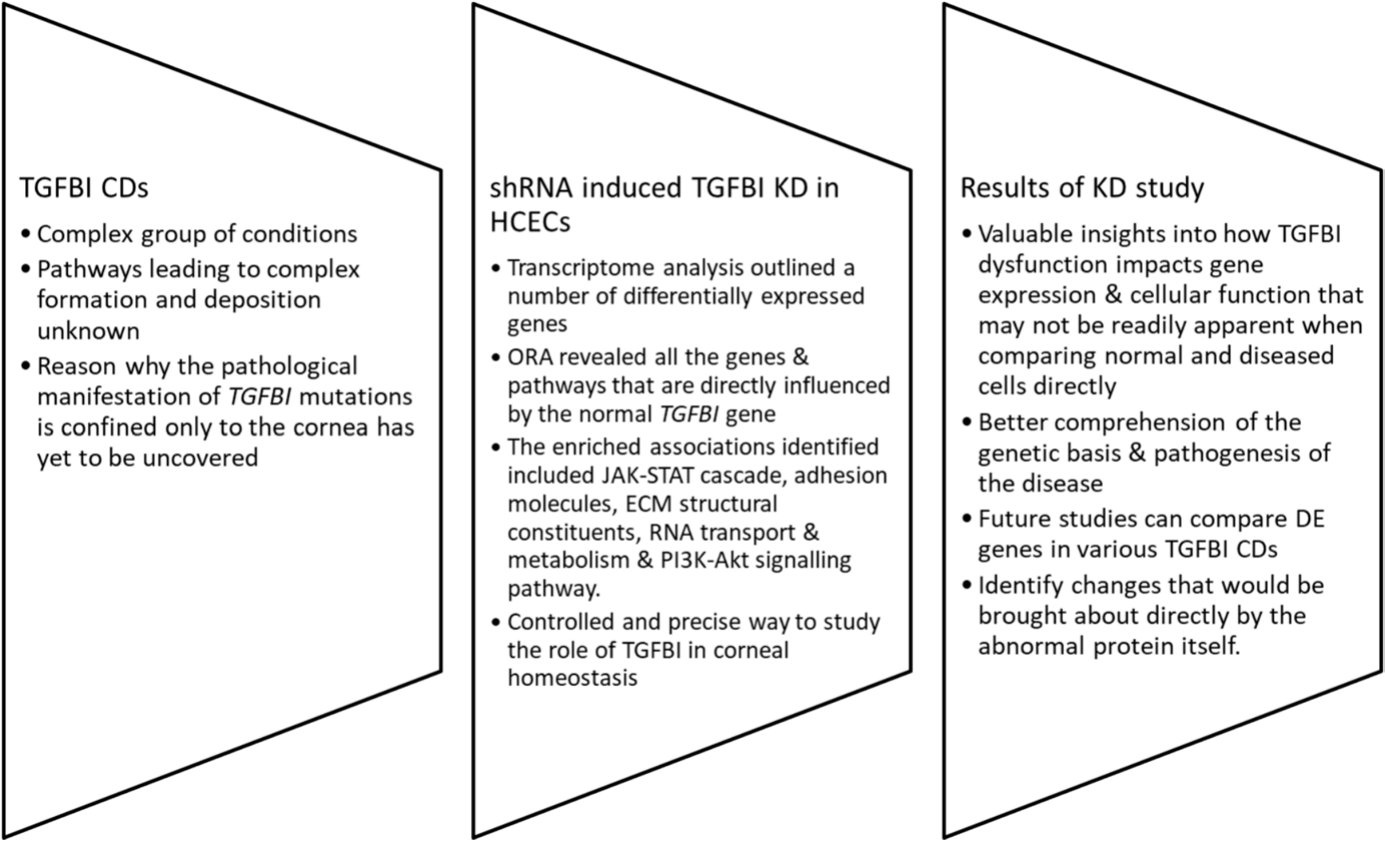
Schematic diagram explaining potential implications of this TGFBI KD study in increasing the understanding of CDs

Our TGFBI KD study establishes a foundational gene expression profile by mapping the genes and pathways directly regulated by normal TGFBIp, shedding new light on how *TGFBI* dysfunction impacts cellular function, thus leading to a better comprehension of the pathogenesis of disease. One has to keep in mind that even though a number of expression changes were identified at the molecular level, the actual side effects these observed changes might have at a functional level in the cornea require further verification in future studies (Figure 7). Additionally, by comparing these findings with transcriptomic data from *TGFBI* CD patients with various mutation variants (e.g. R124C, R555W) future research can pinpoint disease-specific changes that would be brought about directly by the abnormal protein itself and would definitely help in outlining the pathophysiology of each specific *TGFBI* dystrophy.

### RNAi in TGFBI Research and Implications for Future Clinical Practices in the Treatment of TGFBI CDs

The breakthrough discovery of shRNA-mediated gene silencing allows for targeted gene knockdown to study gene function in the context of specific cellular pathways. It provides the opportunity of unraveling the uncertainties of individual gene function in cell physiology and disease states as well as in identifying previously unknown interacting genes.

Recent studies have explored the use of RNAi in HCECs to inhibit pathological corneal processes, with most employing siRNAs and fewer utilizing the more stable shRNA approach (Cao et al. 2023; Yellore et al. 2011). Notably, only two studies have reported the use of RNAi to target specifically the *TGFBI* gene in human corneal epithelial cells, and this was done by using siRNA (Courtney et al. 2015). RNAi was applied by (Courtney et al. 2015) to specifically target a LCD mutant allele. While this allele-specific strategy could be applicable to other *TGFBI* mutations, this method would be only effective in heterozygous patients since it still relied on the production of wild-type TGFBIp. Thus, in homozygous patients, one would still have to consider complete *TGFBI* KD – a challenge compounded by the growing number of identified *TGFBI* mutations that have been attributed to various phenotypes of *TGFBI*-associated corneal dystrophies. Therefore, preferably, treatment plans that target all the subtypes in the *TGFBI* CD group would be desirable by aiming to suppress the disease-causing protein.

Yellore et al. (2011) investigated the efficacy of siRNA in decreasing TGFBIp expression in TGF-*β*1-induced and non-induced HCECs, and concluded that TGFBIp production in HCECs can be inhibited with RNA interference. These studies provided insight into the potential of partial or complete knockdown of *TGFBI* as a future therapeutic strategy to treat *TGFBI*-linked CDs. However, the effect on corneal matrix structure and potential consequences on corneal homeostasis when reducing or stopping TGFBIp production in the cornea had not been explored (Poulsen et al. 2018). Subsequently, in order to investigate the possible side effects of total KD of *TGFBI* on corneal integrity, Poulsen et al. (2018) analyzed the histological structure of corneas in TGFBI-deficient mice and reported that even after eliminating TGFBIp totally from the mouse cornea, the mouse cornea still exhibited structural integrity with only minor structural changes seen.

The results obtained from such research are encouraging, however, given that mice express much lower corneal TGFBIp levels when compared to humans, further studies specifically targeting HCECs are essential. Besides, even though Poulsen et al. (2018) ventured along the path of investigating the corneal proteomic profiles in TGFBI-null mice, none of these projects looked into the downstream effects *TGFBI* KD could subsequently have on human corneal epithelial cell pathways and the expression of *TGFBI* related genes, as opposed to mice corneas. Thus, the potential side effects of knocking down one of the most abundant proteins present in the human cornea still remain unclear and definitely worthwhile being explored.

Our research addresses this gap and provides critical insights into the consequences of silencing this abundant corneal protein with multiple physiological roles.

RNAi-based gene therapy approaches have opened up new horizons for patients with limited treatment options (Du and Palczewski 2023). This discovery led to the development of a new class of medicines that are ideal for conditions that have a genetic component associated to them.

Medications to treat eye conditions can be administered both topically and systemically. By far, the commonest and least invasive route for the treatment of anterior segment conditions is topical application (Kitamoto et al. 2020). The distinctive anatomical and physiological features of the corneal epithelium make it a particularly appealing target for non-invasive topical genetic therapy, especially due to its avascular nature and its immune-privilege. Having said that, low ocular bioavailability can decrease their efficacy due to the presence of multiple physiological barriers that are present in the cornea. To mention a few: the limited surface area of the cornea, reflex blinking that removes most of the volume applied, the tight junctions between the hydrophobic epithelial cells and the hydrophilic stroma of the cornea, all impede the passive diffusion of drug molecules (Gaudana et al. 2009). Furthermore, the unwanted absorption of the drug systemically can result in adverse effects. Recent advances in nanotechnology have contributed to the development of new viral and non-viral drug delivery systems (such as liposomes, compacted nanoparticles, and electroporation among others) to help counteract these issues while minimizing inflammatory and immune responses (Jumelle et al. 2020). Researchers have also embarked on developing an effective, safe, and non-invasive means of delivering RNAi to the cornea. These include hybrid silicon-lipid nanoparticles, modified cell-penetrating peptides, and pH-sensitive vehicles among others (Cao et al. 2023; Baran-Rachwalska et al. 2020; SchiroLi et al. 2019).

**Fig. 8:**
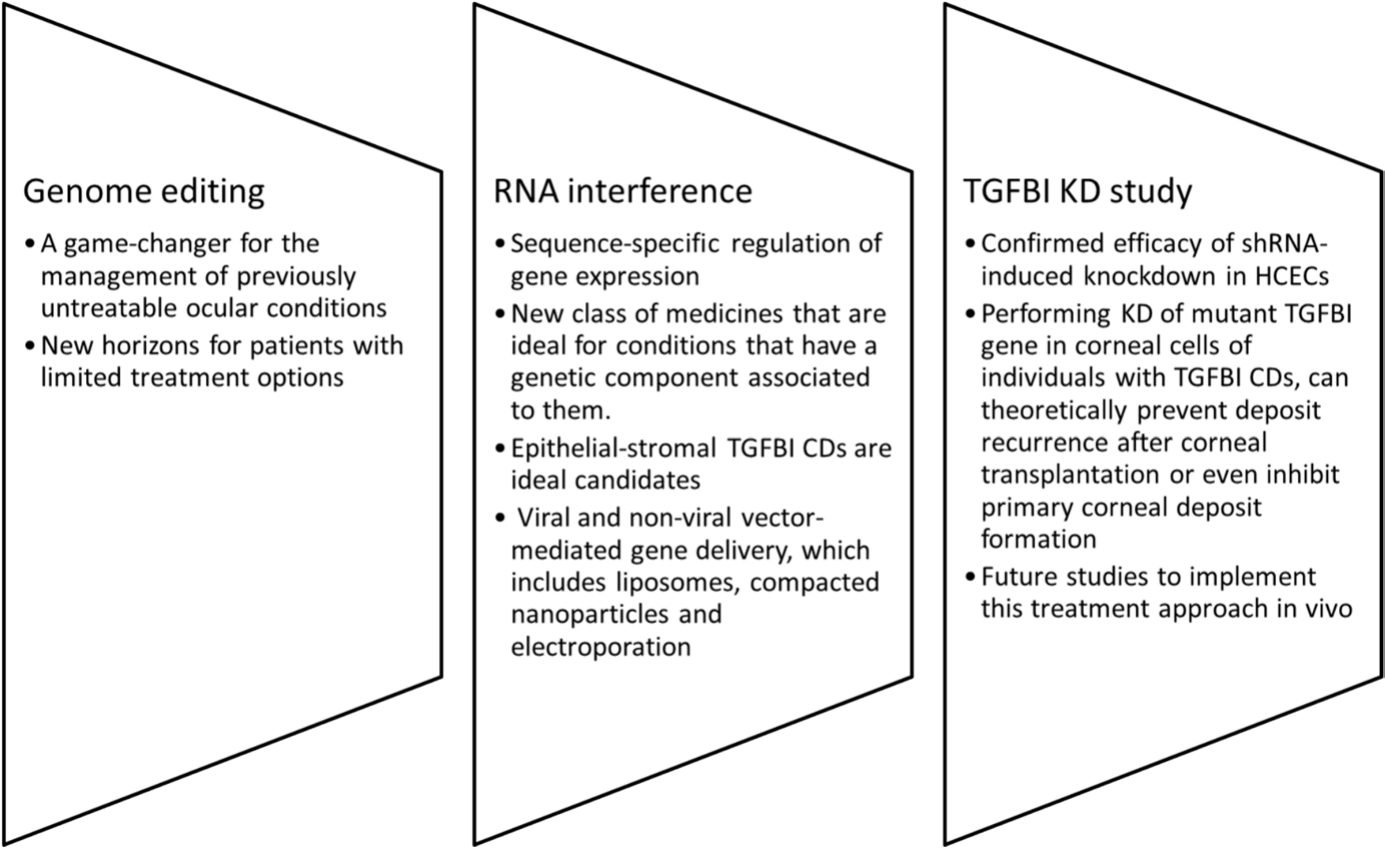
Schematic diagram explaining the implications of this TGFBI KD study for future clinical practices in the treatment of TGFBI CDs

Silencing mutant *TGFBI* in individuals exhibiting *TGFBI* CDs can potentially prevent protein deposition post-transplantation or even halt primary deposit formation, addressing the root cause of the disease. This study demonstrated successful *TGFBI* KD in HCECs using shRNA, suggesting its potential as a treatment for *TGFBI* CDs, hence paving the way for future studies to implement this treatment approach in vivo (Figure 8).

### Limitations

*TGFBI* is also known to act as a tumor suppressor gene, and, together with the upregulation of *AKT1* (a known oncogene) seen in this study, the possibility of oncogenic consequences must be addressed. Tissue-specific delivery to corneal epithelial cells could mitigate this concern, as corneal neoplasms are rare (Rao and Shields 2019). Furthermore, interestingly, (Poulsen et al. (2018) documented only minor changes in corneal matrix composition of *TGFBI*-null mice. These results should reasonably be analyzed with caution when extrapolating them to humans, since, as correctly pointed out by the authors themselves, human corneas express higher TGFBIp levels than mice (Poulsen et al. 2018).

A few limitations of shRNA technology must be mentioned. Unfortunately, the complete evasion of off-target effects caused by shRNA gene silencing is probably impossible; however, in this study these were minimized as much as possible. Off-target effects can arise due to nucleotide sequence homology with non-target transcripts, anomalous processing of endogenous miRNAs and disruption in cell homeostasis owing to the large presence of exogenous vectors and RNAs (Fellmann and Lowe 2014). Off-target effects due to sequence based homology were minimized by a) identifying shRNAs that are likely to target non-target transcripts by employing the BLAST function in NCBI and b) experimentally by using multiple shRNAs (Jackson and Linsley 2010). Aberrant processing of endogenous miRNAs can also lead to off-target effects due to saturation of miRNA processing by Dicer complexes (Gu et al. 2012), experimental and endogenous RNAi competing for incorporation in RISC as well as unintended guide sequences being produced after processing of shRNA precursors (Fellmann and Lowe 2014; Kawahara et al. 2007).

Other known limitations of shRNA include the potential to trigger interferon or other immune responses, cellular toxicity and variable silencing duration (Goel and Ploski 2022). While transient delivery may result in short term silencing, stable integration can lead to genomic instability. ShRNA can provide long-term gene silencing, however, its long-term safety depends on the expression levels, delivery methods, and potential off-target effects. These can be addressed by controlling promoter strength, using miRNA-adapted shRNA scaffolds to reduce immune activation and using improved delivery systems (Kwak et al. 2019).

RNA-seq can be used in various analysis strategies that include transcript analysis of samples of organisms with known, well annotated genomes or even analysis of samples of organisms without known sequenced genomes. However even though it is very sensitive and can measure gene expression levels accurately, results can be affected by the challenging process of library preparation, sequencing depth, and bioinformatics analysis pipelines.

Protein-level confirmation, such as Western blot or immunofluorescence would provide an added layer of validation, however the primary objectives of this study were to: (1) delineate the transcriptomic landscape following TGFBI suppression and (2) explore the feasibility of shRNA-mediated gene silencing as a therapeutic concept. The observed changes in differentially expressed genes across key pathways (e.g., SMAD, JAK-STAT, PI3K-AKT) indicate robust transcriptional perturbation in response to TGFBI KD, offering biologically meaningful insights into its role in corneal homeostasis. Future follow-up studies will include protein-level quantification to fully assess translational impact, especially in the context of preclinical and in vivo applications.

In summary, RNAi-based *TGFBI* silencing holds promise for treating *TGFBI* CDs, but further research is needed to optimize delivery and assess long-term safety before clinical translation.

## Conclusion

To the best of our knowledge, this study is the first comparative transcriptomic analysis that explores the differential expression of genes in *TGFBI* KD HCECs using RNA-seq generated gene expression data. It sheds new light on the key genes that are associated with *TGFBI* and provides us with a skeleton of *TGFBI*-associated pathways, thus, delineating the physiological role of TGFBIp in the corneal epithelium. It provides a valuable insight into how *TGFBI* dysfunction can impact corneal function, which might not be readily apparent when comparing normal & diseased cells directly.

ORA performed on DE genes from RNAseq results of the TGFBI KD HCECs identified enriched associations that included adhesion molecules, extracellular matrix structural constituents, cell cycle regulation, RNA transport and Metabolism, the JAK-STAT cascade, the FoxO signaling pathway, and the PI3K-Akt signaling pathway. Even though a number of expression changes were identified at the molecular level, the actual side effects these changes might have at a functional level in the cornea might be minimal.

In view of the unmet clinical needs and treatment options available for the management of *TGFBI* CDs, *TGFBI* inhibition in HCECs as a possible genetic therapeutic approach was also explored. This study leads us to conclude that shRNA-mediated gene KD in HCECs is effective and shows strong potential for subsequent future in vivo studies, with the aim of finally implementing this method as a novel treatment for *TGFBI* CDs.

## Data Availability

The data that support the findings of this study are openly available in the github repository at https://github.com/gabysci/TGFBI_KD_HCEC_VS_CONTROL
